# Contextual Modulation of Biases in Face Recognition

**DOI:** 10.1371/journal.pone.0012939

**Published:** 2010-09-23

**Authors:** Fatima Maria Felisberti, Louisa Pavey

**Affiliations:** Faculty of Arts and Social Sciences, Kingston University London, London, United Kingdom; Kyushu University, Japan

## Abstract

**Background:**

The ability to recognize the faces of potential cooperators and cheaters is fundamental to social exchanges, given that cooperation for mutual benefit is expected. Studies addressing biases in face recognition have so far proved inconclusive, with reports of biases towards faces of cheaters, biases towards faces of cooperators, or no biases at all. This study attempts to uncover possible causes underlying such discrepancies.

**Methodology and Findings:**

Four experiments were designed to investigate biases in face recognition during social exchanges when behavioral descriptors (prosocial, antisocial or neutral) embedded in different scenarios were tagged to faces during memorization. Face recognition, measured as accuracy and response latency, was tested with modified yes-no, forced-choice and recall tasks (*N* = 174). An enhanced recognition of faces tagged with prosocial descriptors was observed when the encoding scenario involved financial transactions and the rules of the social contract were not explicit (experiments 1 and 2). Such bias was eliminated or attenuated by making participants explicitly aware of “cooperative”, “cheating” and “neutral/indifferent” behaviors via a pre-test questionnaire and then adding such tags to behavioral descriptors (experiment 3). Further, in a social judgment scenario with descriptors of salient moral behaviors, recognition of antisocial and prosocial faces was similar, but significantly better than neutral faces (experiment 4).

**Conclusion:**

The results highlight the relevance of descriptors and scenarios of social exchange in face recognition, when the frequency of prosocial and antisocial individuals in a group is similar. Recognition biases towards prosocial faces emerged when descriptors did not state the rules of a social contract or the moral status of a behavior, and they point to the existence of broad and flexible cognitive abilities finely tuned to minor changes in social context.

## Introduction

Faces are salient and highly relevant visual stimuli to social interactions [1], [2], [3] and trait judgments of others can be made in less than a second and from a minimal amount of information [4], [5]. Hence, face recognition skills are developed from early childhood onwards and rely on recollection and familiarity to judge whether a face seen presently has occurred earlier [6], [7]. Research examining the cognitive processes involved in social exchange finds that across a broad array of exchanges, individuals displaying prosocial (*e.g.* cooperation) and antisocial (*e.g.* cheating) behaviors are tagged with symbols or labels, which are later used to decide about approachability or avoidance, trust or distrust [8]. One function of prosocial behavior is that it enhances group cohesion, which provides individual benefits to group members by increasing individual well-being, maximizing gains, and increasing safety and security for individuals existing within that group. Cooperation is associated with greater group identification [9], loyalty [10] and trust [11]. Antisocial behavior however, serves to undermine group cohesion [12], and is usually punished through social exclusion [13]. According to Cosmides, Barrett and Tooby [14], cheaters take advantage of a social contract by *intentionally* failing to share its cost and, therefore, need to be detected early to avoid exploitation. Reputations are also remembered such that ‘second order’ rewards occur in the form of support for those who favor cooperation [15].

Social exchanges such as tit-for-tat and reciprocal altruism rely on cooperation for mutual benefit, and are suggested to operate via a rational choice model based on economic principles of costs and gains analysis. The theory of reciprocal altruism, [16] suggests that cooperation can evolve when people are able to identify cheaters and redirect their prosocial behavior towards cooperators who are likely to reciprocate [17]. Further, reputations for cooperative behavior have been shown to advance social status [18] allowing competitive altruism to emerge [19]. However, prosocial behaviors pose an interesting dilemma for social and evolutionary psychology researchers, who in the 1970's and 1980's started using models to uncover the conditions necessary for cooperation to occur. Results from models based mainly on the Prisoner's Dilemma game and a computer tournament offered some glimpses into how cooperation based on reciprocity may start, thrive and finally succeed in an asocial environment [20].

Some researchers proposed that humans may be equipped with an altruism-detection mechanism [21]. In a zero-acquaintance video presentation paradigm, for example, participants were able to accurately detect altruists just by looking at certain recorded facial expressions [22]. In addition, enhanced signal changes in the face-processing area of the fusiform gyrus have been recorded during trustworthiness judgments [23], [24]. Cosmides and Tooby [25], [26], conversely, argue that in order to engage successfully in social exchanges, the architecture of the human brain evolved to include modular cognitive abilities to solve a number of complex problems, embracing a powerful mechanism to detect cheaters. In support of their computational social theory of social exchange, biases in recognition have been reported with faces associated to behaviors categorized as antisocial [27], [28]. Mealey, Daood and Krage [29] found an enhanced memory for faces tagged with descriptions indicating cheating or potential threat and Chiappe, Brown, Dow, Koontz, Rodriguez, and McCulloch [30] showed that accuracy was higher and gaze latency was longer for faces of cheaters. Adding to the complexity of face recognition in scenarios of social exchange, recent experiments have reported no reliable biases for faces of cheaters or cooperators [31], [32].

**Figure 1:**
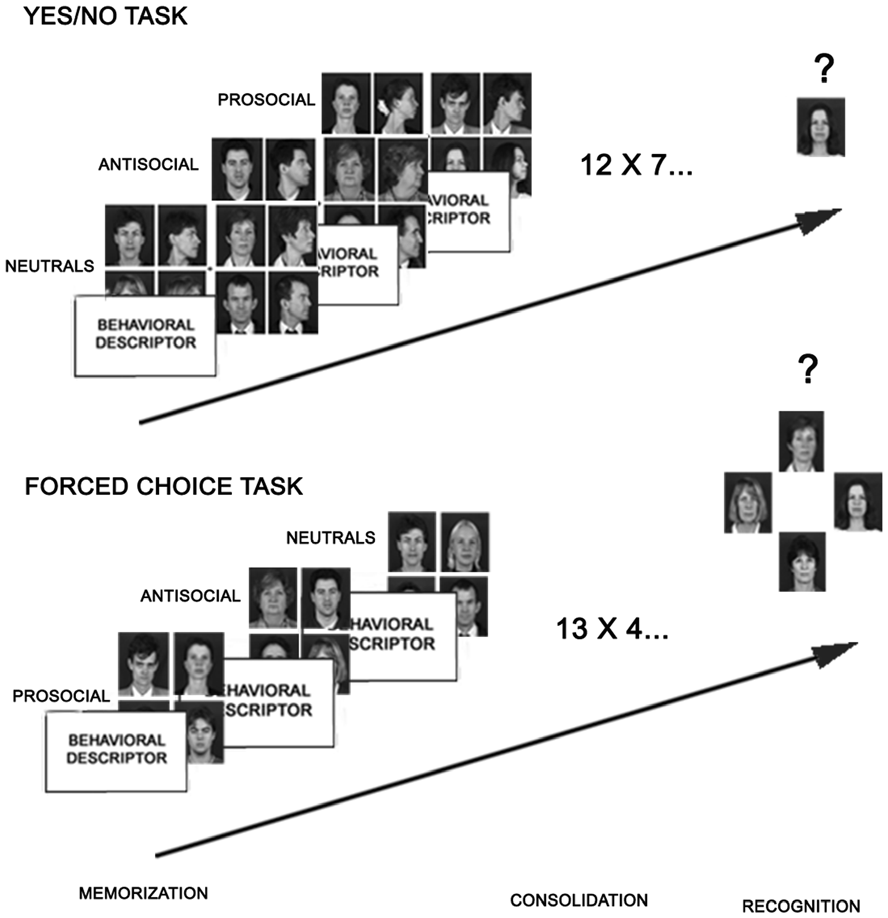
Schematic representation of procedures used in the study. Participants had to memorize three groups of faces. A screen with a behavioral descriptor appeared before the presentation of each group of faces (prosocial, antisocial and neutral). The order of descriptors and correspondent group of faces was randomized and counterbalanced. Memorization was followed by a distracter task (consolidation) consisting of a series of multiplications. In the “yes/no” task each group had 4 faces and each face appeared in frontal and profile view (8 images/group; frontal view always to the left of its profile view). Participants had to answer whether they had seen the displayed face before (50% tagged faces and 50% new faces). In the 4-alternative forced-choice task each group also had 4 faces, but only in frontal view. In this task participants had to choose which of the faces they had memorized (25% tagged faces, 75% new). The display for the recall task was similar to the yes/no task, but participants had to answer whether the face they saw belonged to “cheaters”, “neutrals”, or “cooperators” instead (only tagged faces were presented). Tagged and new faces were interleaved and presented randomly.

Successful human social exchanges depend, to a large extent, on accurate identification and trait judgments of group members. According to Bayley, Wixted, Hopkins, and Squire [33], recollection entails remembering specific details about the event in which a face was encountered, while familiarity entails simply knowing that a face was seen before, even when no contextual information can be retrieved. It has been proposed that recollection and familiarity could be assessed differently by using the yes-no and forced-choice procedures, respectively [34], [35]. Accordingly, in a forced-choice task individuals discriminate memorized faces from new ones on the basis of relative familiarity (i.e., individuals see two or more faces and have to choose the familiar/memorized one), while on a yes-no task, successful performance involves some degree of recollection (i.e., individuals only see one face and have to decide if it is familiar or not) [33] (Figure 1).

The experiments described here use three different recognition tasks (modified yes-no, four-alternative forced-choice and “classical” recall) to examine the extent to which face recognition can be affected by different encoding scenarios and related moral status (referred to as behavioral descriptors), when the number of prosocial and antisocial faces to be encoded is the same. Face recognition was tested in four experiments under two social scenarios (financial transaction or social judgment) and variations in the behavioral descriptors (prosocial, antisocial or neutral) tagged to faces during encoding. The impact of making moral behaviors explicit prior testing was also investigated.

## Methods

All experiments had a similar core structure: (i) encoding (memorization of behavioral descriptions and faces), (ii) memory consolidation (distracter task: simple multiplications), and (iii) face recognition tasks. The within-subject independent variable was the category of descriptors (antisocial, prosocial, neither, referred to as neutral) and the dependent variables were the accuracy and the response latency to correct responses. Partial *η^2^* is reported as an effect-size measure.

### Participants

Participants, all undergraduate students (*N* = 174), were recruited via internal mail and provided written consent in accordance with procedures approved by the Ethics Committee of the Faculty of Art and Social Sciences (Psychology Unit) at Kingston University and in accordance with the ethical guidelines of the British Psychological Society. Some students received bonus course credits, but no financial compensation for participation in the experiments was given. All participants had normal or corrected-to-normal vision and were between 18 and 38 years old (modal age = 21; some declined to give their age).

### Materials

Photographs of an equal number of males and females were taken from University College London XMT2VS database (227×181 pixels). Both frontal and profile head-shot photographs were used. In the pictures, all “actors” stood against a dark blue background and had neutral expressions. At approximately 50 cm from the centre of the monitor, pictures had a viewing angle of 6.8×5.5 degrees. E-Prime (Psychology Software Tools, Pittsburgh, PA) was used for stimulus presentation.

### General procedure

After reading and signing the consent forms, participants were told the experiments were about memory for faces, but were not cued about the type of memory task employed. Figure 1 shows a schematic representation of the general procedure. In the encoding phase a screen introduced the social context, referred to as scenario (see below), before presenting a screen with one of three categories of descriptors of moral behavior (prosocial, antisocial or neutral). Each descriptor preceded a screen with a group of 4 faces (50% males, 50% females) in frontal and profile views (8 pictures, experiments 1 and 3) or in frontal view only (4 pictures, experiment 2). Each group in experiment 4 contained 6 faces, all in frontal view. Profile views were introduced to test the strength of memorization since there are suggestions that familiarization with frontal and profile views enhance face recognition [36]. The order of group presentation was randomized. The time to read the descriptors was unlimited, but the duration of face encoding was 6 sec for each group in experiments 1 and 3 and unlimited for groups in experiments 2 and 4. Willis and Todorov [4] showed that just 100 msec of exposure to a neutral face was enough for judgments about trustworthiness and aggressiveness, for example.

The encoding phase was followed by the consolidation phase, which consisted of a series of multiplications that lasted 3–5 min, with answers entered with the keyboard and feedback provided.

This phase was followed by the face recognition tasks, described in more detail for each experiment in the appropriate section (cf. Fig. 1). A screen with the instructions related to a given task appeared and participants pressed a key to continue when ready. Participants were then asked to respond to the recognition task as quickly and accurately as possible. Then a black fixation cross was presented on a blank screen for 1 sec before the task started. The trial order was randomized and feedback was provided. Note that descriptor and group of faces were kept constant in experiments 1 and 3. Each group of faces was the same for all participants in experiment 1. Faces in each group were then changed in experiment 2 (50%), experiment 3 (25%), and in experiment 4 (1/3) in relation to experiment 1. Additional control experiments for the faces used in this study are described in Results.

### Scenarios

#### Scenario 1 (financial loan)

Participants were given a scenario adapted from Chiappe and colleagues [30]. The introductory screen read: “Before you continue, it is important to know that John is a successful businessman. Through his hard work, he has managed to build a very good life for himself and his family. He is also quite generous. He is willing to help out his long-time friends by offering them loans when needed. In the next 3 screens you will meet John's friends. Press any key to continue.” The next screen read: “You will see 3 groups of people. The groups have different behaviors, which are specified in a screen before their photos. Press any key to continue”. Then, before being shown the faces, prosocial, antisocial or neutral behavioral tags were introduced. The content of the different behavioral tags was: (prosocial) “This group of friends borrowed £25,000 from John and paid it back with interest within a year”; (antisocial) “This group of friends borrowed £25,000 from John and never paid it back”; (neutral) “This group of friends never borrow money from John”.

Note that the template for the conditional rule is of the form: “if you take benefit P, then you must satisfy condition Q”, but unlike Chiappe and colleagues experiment, the descriptors here did not specify the social contract rule nor mention the words “cheater” or “cooperator”.

#### Scenario 2 (social judgment)

The first screen contained the following instruction: “You will see 3 groups of people. The groups have different behaviors, which are specified in a screen before their photos. Press any key to continue”. The behavioral tags were: (prosocial) “The people you will see in this set have: donated £10,000 to charity, worked with children in Africa, helped elderly people, fostered over 10 children, raised over £1,000 by running a marathon”; (antisocial) “The people you will see in this set have committed some illegal actions: sold over 1000 illegal DVDs, drove whilst disqualified, invaded a football pitch, committed major benefit fraud, kidnapped a young woman”; (neutral) “The people you will see in this set have different hobbies: shopping at Tesco, swimming and walking, driving fancy cars, eating out”. Again, descriptors did not specify the social contract rule nor mention the words “cheater” or “cooperator”, but used instead words strongly linked to prosocial (e.g., donated, charity, helped, fostered), antisocial (e.g., illegal, invaded, fraud, kidnapped) and neutral behaviors (e.g., hobbies, shopping, swimming, eating out), confirmed in a pilot experiment with six participants.

### Analysis

Average mean accuracy and response latency, also referred to as reaction time (RT), were subjected to a repeated-measures ANOVA with four behavioral contexts: prosocial, antisocial, neutral and new (untagged faces)×2 angles of view (frontal vs. profile) as the factors. The recall task had only the first three categories. Accuracy or RT values used in the statistical analysis represented the average of 144–192 trials, *i.e.* 36–48 trials per condition/participant. Participants with overall accuracy below 60% (*i.e.* (prosocial+antisocial+neutral+new)/4) in a yes-no recognition task were eliminated from the analysis, as their overall performance was too close to chance level (*N* = 15). In the recall task no participants were eliminated (chance level around 33%). Greenhouse-Geeiser adjustments to the degrees of freedom were performed when sphericity could not be assumed (Mauchly's sphericity test). All pairwise comparisons were carried out with Bonferroni adjustments.

## Results

### Control Experiment

The baseline accuracy and response latency, referred to as RT, for face recognition was established with two control experiments. In each experiment an introductory screen read simply: “The next screen shows some of John's friends. Press any key to continue”. Then three other screens were presented, each one of them containing 6 faces to be memorized in the absence of an encoding scenario; no behavioral descriptors were tagged to the faces. For more details about the retention and recognition tasks see General Methods.

The first recognition task (*N* = 10, 8 women, 2 men) was a modified yes-no task, which consisted of a single face presented in the middle of the screen and participants had to answer whether they had seen the face in the memorization phase or not by pressing “1” for YES and “2” for NO (50% tagged faces and 50% new faces). Note that this is a modified yes-no recognition task, in that not only each of the encoded faces were presented 3 times (i.e. 3 cycles of trials), but also each of the new, non-tagged faces. Therefore, in the first cycle of trials, participants simply had to remember the memorized, tagged faces and which were the new, untagged faces. In the remaining two cycles of trials, the task became harder since by now all faces became “familiar”, either because they had been encoded during the memorization phase or because they had been presented in the first (and second) cycles of trials.

The second recognition task (*N* = 13; 11 women, 2 men), the four-alternative forced-choice task, consisted of four faces displayed around an imaginary circle at the centre of the screen: one face belonged to one of three tagged groups, while the other three faces were new. Participants had to choose the location of the tagged face (left, right, top, or bottom) by pressing designated keys in the keyboard. Faces were presented in each of the four positions and the order of the presentation varied randomly across trials.

In the absence of behavioral information during face encoding, no significant differences in accuracy were observed between the groups of untagged faces in the two experiments, *F*<1. Overall accuracy in the modified yes-no (*M* = 93%, *S.E.* = 2) and in the forced-choice (*M* = 89%±*S.E.* = 3) recognition tasks was similar and no significant differences in RT were observed (2381±205 msec and 2138±162 msec, respectively). Accuracy to the 18 individual faces was also similar, *F*(17,22) = 1.20, *p* = .26. Table 1 shows the accuracy and reaction times of obtained in all experiments described here.

**Table 1:** Accuracy (%) and reaction time (msec) for the recognition and recollection of faces in frontal view and tagged with descriptors of antisocial, prosocial, or neutral behaviors (*mean* ± *SE*).

	Accuracy (%)			
	Antisocial	Prosocial	Neutral	New
**Experiment 1**				
yes-no task	63±3	85±3**	67±3	75±3
recall task	43±3	53±3*	36±3	
**Experiment 2** (*forced-choice*)	88±3	92±2**	85±3	
**Experiment 3**				
yes-no task *(questionnaire+descriptors)*	79±3	78±3	76±3	72±3
yes-no task *(short descriptors only)*	74±3	84±3	77±3	75±3
**Experiment 4**				
yes-no task	78±2*	83±2*	70±2	79±2
recall task	51±3**	50±3**	34±3	

	Reaction Time (msec)			
	Antisocial	Prosocial	Neutral	New
**Experiment 1**				
yes-no task	1036±49	860±38**	948±41	1034±53
recall task	1156±128	1053±96	1416±111	
**Experiment 2** (*forced-choice*)	1877±81	1809±64	1911±67	
**Experiment 3**				
yes-no task *(questionnaire+descriptors)*	1270±66	1138±45	1393±57	1316±55
yes-no task *(short descriptors only)*	1094±46	1009±57	1132±58	1225±74
**Experiment 4**				
yes-no task	975±31*	931±29*	1053±35	1061±31
recall task	1927±76*	2478±141	2265±118	

*p<.05, ANOVA.

**p<.001, ANOVA.

Next to experiment number is the type of recognition task.

### Experiment 1

This experiment was designed to test whether brief behavioral information tagged to faces in a financial encoding scenario (*i.e.*, borrowing £25000 and paying it back or not) would lead to face recognition biases when using a set of different recognition tasks. This experiment used a scenario adapted from Chiappe and colleagues (2004), where the moral status of the behavioral descriptors was made explicit by using the words “cheaters” or “cooperators”. In this experiment, however, descriptors simply described a behavior and were not accompanied by words making their moral status explicit. The aim was to investigate whether the omission of such explicit terms would affect face recognition.

#### Method

There were 26 participants (18 women, 8 men), all university students. Scenario 1 (financial) was used. The experiment had 24 faces from 12 actors (50% frontal and 50% profile views) and each of the three behavioral categories had 8 faces (from 4 actors) to be memorized.

Two recognition tasks were employed: a modified yes-no task and a recall task. The modified yes-no task was introduced by a screen with the instruction: “Have you seen this face before?” Press: 1 = yes, 2 = no. The instruction for the recall task was: “Is the face you see linked to cooperators, cheaters or neutral behaviors?” Press: 1 = cooperators, 2 = cheaters, 3 = neutrals. Note that participants had to equate the prosocial descriptor with “cooperators” and the antisocial descriptor with “cheaters”.

After reading the instructions and pressing a key to continue participants saw a black fixation cross on a blank screen (1 sec), followed by a face (tagged or new one), which remained on the screen until one of the possible responses to the given task was selected and entered with the keypad.

#### Results

**Figure 2:**
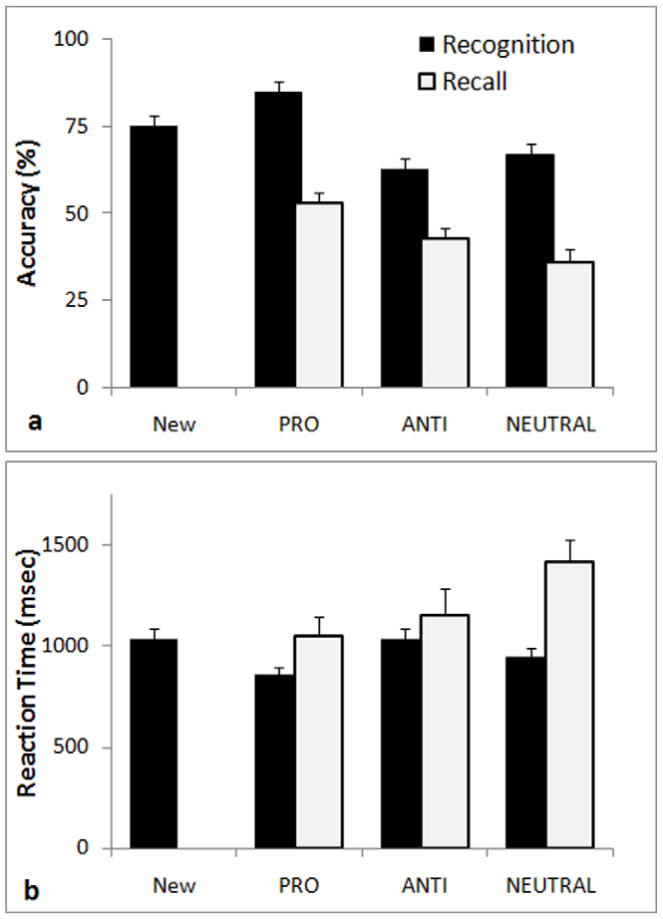
Accuracy (a) and reaction time (b) for recognition (blank columns) and recall (white columns) of faces tagged in a financial scenario with brief descriptions associated with antisocial (ANTI), prosocial (PRO), neutral (NEUTRAL) behaviors or New faces (error bars show +*S.E.*).

Modified yes-no task: The repeated presentation of new, untagged faces alongside tagged faces instead of a unique presentation of each face in early studies may have lead to a slightly higher level of errors, but trial repetition conferred robustness to the averaged accuracy values reported here. Results showed a significant recognition bias to tagged faces, *F*(3,75) = 12.44, *p*<.001, *η* ^2^ = .33 (Table 1). An enhanced recognition of prosocial faces was observed in comparison to antisocial, neutral and new faces (*p*<.02; Figure 2a). Recognition of antisocial and neutral faces was similar, but there was an interaction between tags and viewing angle, *F*(3,111) = 6.10, *p*<.001, *η* ^2^ = 0.14. Accuracy for antisocial and neutral faces in frontal view was higher than when in profile view, *F*(3,37) = 13.46, *P* = 0.001, *η* ^2^ = 0.27, but still lower than for cooperators, *p*<.001. RT also varied with tags, *F*(3,75) = 9.40, *p*<.001, *η* ^2^ = .27 (Figure 2b). RT for prosocial faces was shorter than for antisocial, neutral, and new faces. There were no significant differences in latencies related to viewing angle, *F*(1,37) = 3.03, *p* = 0.09. Part of the results related to profile views of the tagged faces was presented at the Conference of the European and Human Behaviour Association [37].

Recall task: Fourteen participants run the recall test straight after the recognition test. As expected, recall also varied with behavioral tags, *F*(2,32) = 7.86, *p* = .002, *η* ^2^ = .33 (Table 1). Accuracy rates for recall (three-alternative forced choice task; chance level at 33%).were lower than for recognition. Again, mean accuracy for prosocial faces was higher than for antisocial and neutral faces (*p*<.02; Figure 2b). The enhanced recognition of prosocial faces was observed with faces in frontal and in profile views. There was also a significant difference in RT, *F*(2,32) = 4.22, *p* = .024, *η* ^2^ = .21, with neutral faces demanding more time for correct recall than prosocial and antisocial faces.

#### Discussion

In agreement with the result of Chiappe and colleagues' [30], we also found a longer RT for faces of cheaters, but unlike their results showing a better recognition of cheaters, we found that prosocial faces were recognized and recalled better and quicker than antisocial or neutral faces. The higher accuracy rates for prosocial faces cannot be explained by familiarity alone as participants were able to recall the behavioral context tagged to the faces. In addition, results showed that high prosocial accuracy was obtained with shorter response latencies.

The absence of an explicit warning about the presence of cheaters in the descriptors tagged to faces might explain the relative lower accuracy to cheaters in this experiment. The ability to recognize faces of cooperators accurately is advantageous, as it allows us to approach them in future instances of exchange and avoid cheaters [22], [38]. In line with this findings, Price [39] reported that people displayed a tendency to favor more cooperative workers, while Oda, Hiraishi and Matsumoto-Oda [40] suggest that an independent altruist-detection algorithm would be activated when a relationship of social exchange with another person has not been established. Once social exchanges have occurred, a cheater-detection mechanism would be activated to maintain the relationship. In other words, the ability to detect altruists and cooperate exclusively with them would reduce the probability of exploitation in social interactions [21].

### Experiment 2

Bastin and Van der Linden [34] proposed that recollection and familiarity can be assessed differently by using the yes/no and forced-choice procedures. As mentioned previously, in a forced-choice task individuals discriminate memorized faces from new ones on the basis of relative familiarity (i.e., they see two or more faces and have to choose the familiar one), while on a yes-no task, successful performance involves some degree of recollection (i.e., individuals only see one face and have to decide if it is familiar or not) [33]. The aim with this experiment was to investigate if the recognition biases observed in experiment 1 using the yes-no and the recall tests would be still present if faces were presented alongside distracter faces in a four-alternative forced-choice recognition task, a likely scenario in many social exchanges. If the recognition bias for prosocial faces observed in experiment 1 was strong and reliable, it should also be observed in a four-alternative forced-choice recognition task.

#### Method

This experiment also used the financial scenario (*N* = 29; 24 women, 4 men). The recognition task employed the four-alternative forced-choice task (cf. control experiment). The task was introduced by a screen with instructions about how to proceed in the test phase: “Choose the face you have seen before by pressing the key correspondent to its location on the screen”, y = top, b = bottom, g = left, h = right. Tagged faces were presented in each of the four positions and the order of the presentation also varied randomly across trials. Then a black fixation cross was presented on a blank screen for 1 sec and it was followed by four faces (all in frontal view); one face always belonged to the memorized set and 3 faces were new ones. Trials were randomized and feedback was given.

#### Results

The recognition biases towards prosocial faces observed in Experiment 1 was confirmed with a different paradigm, *F*(2,56) = 5.23, *p* = .008, *η^2^* = .16. Accuracy to prosocial faces was higher than to neutral and antisocial faces (*p*<.001). No significant differences in RT were observed, *F*(2,56) = 2.58, *p* = .084 (Table 1).

#### Discussion

The results obtained with this task confirmed the prosocial face bias observed in experiment 1 with the modified yes-no and the recall tasks. Sometimes faces can be clearly familiar, but some can fall in a “grey area” forcing the use of whatever information is available for proper evaluation [41]. Therefore, if this recognition task was purely measuring familiarity, one would expect equal performance to all tagged faces.

### Experiment 3

The behavioral tags linked to faces did not contain the terms cooperators, cheaters or neutrals. The lack of an explicit warning to the presence of a cheating/defective behavior during encoding could have favored biases to prosocial faces. The aim of this experiment was to check if priming and/or more concise behavioral descriptors than the ones used in Experiments 1 and 2 would affect face recognition. It was hypothesized that recognition biases would be eliminated by clearly alerting participants to the presence of cheating and cooperative behaviors prior testing.

#### Method

The first part of this experiment (priming and added short descriptors) had 31 participants (16 women, 15 men).

Before the test started participants had to answer three questions on paper:

1. How important do you think it is to remember people who cheated on you?
2. How important do you think it is to remember people who cooperated with you?
3. How important do you think it is to remember people who behaved in an indifferent way to you?

The options for each question were: (a) very important, (b) important, (c) relatively important, (d) not so important, and (e) neutral. The questions aimed to direct the attention to the salient aspects of the encoding condition participants would find in the recognition test that followed.

After filling in the questionnaire, participants run the first part of the experiment. The set up was identical to experiment 1, except for the above questionnaire and a short sentence added to each of the previous behavioral descriptors containing the explicit moral status. In the prosocial descriptor the sentence added was “John judges them as cooperators”; while for the antisocial one the sentence was “John judges them as cheaters” and for neutral it was “John judges them as neutrals”.

The second part of this experiment had 20 participants (18 women and 2 men) as participants. No questionnaires were used and the social reputation contained in the descriptors in experiment 1 was made explicit and contained solely the sentences added in the experiment above (*e.g.*, “John judges them as cheaters”).

#### Results

About 64% of the students judged remembering cheaters very important (36%) or important (28%), while for the majority of them (92%) remembering cooperators was very important (67%) or important (25%). Only 39% of the students considered very important or important to remembering people who ignored them. The results echo the performance observed with face recognition in the previous experiment (Table 1).

Questionnaire, long and short descriptors: The recognition biases observed in experiments 1 and 2 disappeared with this setup, *F*(3,90) = 1, *p* = .36. Recognition accuracy for prosocial faces was reduced from 85% in Experiment 1 to 77%, while accuracy for antisocial faces increased from 63% to 77% and from 67% to 74% for neutral faces. RT was slightly higher than in Experiment 1 and varied with tags, *F*(3,90) = 4.22, *p* = .008, *η* ^2^ = .12. RT for prosocial and antisocial faces was similar, but RT for neutral and new faces was higher than for prosocial faces (*p*<.005).

Short descriptors only: Recognition biases were strongly dampened and accuracy for all tagged faces tended to be more similar than in the priming experiment, *F*(3,57) = 2.68, *p* = .055. The only significant difference in RT was for new faces, *F*(3,57) = 7.72, *p*<.001, *η* ^2^ = .29.

#### Discussion

The similar accuracies for the three behavioral tags when reputations were made explicit, in the presence or absence of priming, point to a more equitable distribution of attentional resources during face encoding. Overall accuracy in these two experiments (about 76%) was lower than the accuracy in the control experiment (about 90%), suggesting that behavioral scenarios during face encoding affected performance. Interestingly, the dampening of accuracy and response latency to prosocial faces in the presence of priming was stronger than with explicit reputations only.

The absence of biases in face recognition found here and in previous experiments [31], [32], [42] could be seen as an indicator of the significance of remembering both cheaters and cooperators. After all, from a natural selection point of view, the only relevant output is the successful identification of cheaters and cooperators with as low a cost and error rate as possible, since such ability is essential for both direct reciprocal cooperation [43], and reciprocity based on reputation [15].

### Experiment 4

According to experiments on reciprocal altruism, an enhanced memory for both prosocial and antisocial individuals exists in order to correctly identify those who deserve to be rewarded with cooperative acts. The last experiments showed that face recognition bias can be modulated by the wording in behavioral descriptors in a social exchange scenario. In the first two experiments, the rules of the social contract and the moral status of the behaviors were not explicitly stated in the descriptors. It is possible that recognition biases may be avoided in a scenario where the described behaviors are strongly associated with cooperation or cheating, even though the rules or moral status are not explicit. This experiment investigates recognition and recall biases using a wider range of descriptors clearly linked to prosocial and antisocial behaviors in each behavioral tag. As remarked previously, descriptors used words strongly linked to prosocial (e.g., donated, charity, helped, fostered), antisocial (e.g., illegal, invaded, fraud, kidnapped) and neutral behaviors (e.g., hobbies, shopping, swimming, eating out), but neither rules of social contract nor the explicit moral status of the behaviors were stated.

#### Method

Fifty seven participants (45 women, 12 men), all university students, took part in this experiment. Scenario 2 (social judgment) was used in this experiment which had 18 faces in frontal view only and each of the three behavioral categories had 6 faces to be memorized. The recognition tasks used in this experiment were the same as in Experiment 1.

#### Results

**Figure 3:**
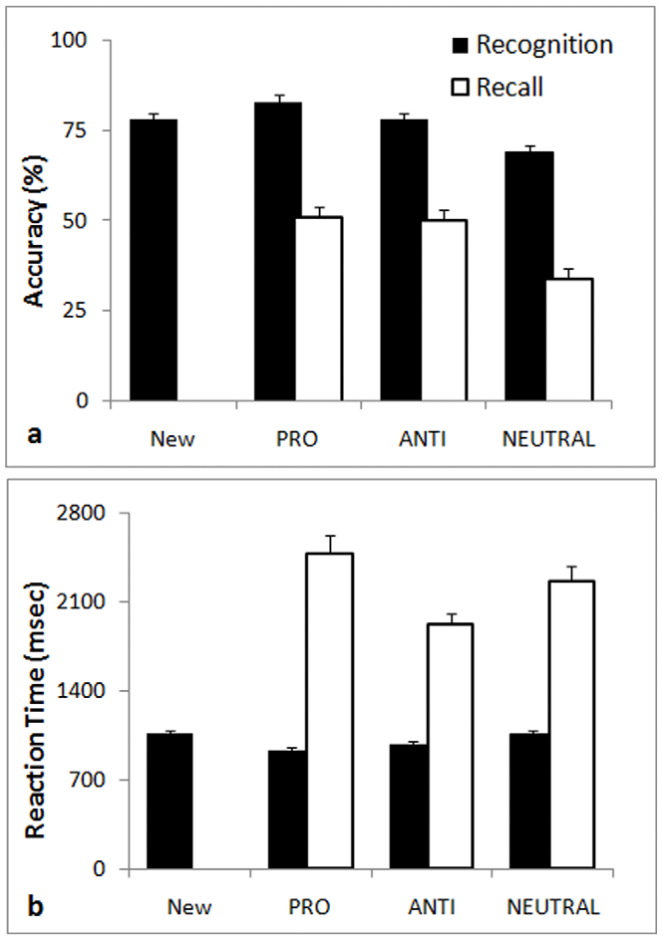
Accuracy (a) and reaction time (b) for recognition (black columns) and recall (white columns) of faces tagged in a social judgment scenario with brief descriptions associated with antisocial (ANTI), prosocial (PRO) and neutral (NEUTRAL) behaviors or New faces (error bars show +*S.E.*).

Modified yes-no task: There was a significant recognition bias to tagged faces, *F*(2.59,152.54) = 13.65, *p*<.001, *η* ^2^ = .21 (Figure 3a). Recognition of prosocial, antisocial and new faces was significantly more accurate than neutral faces (*p* = .004, Table 1). On the other hand, RT varied strongly with behavioral tags, *F*(2.66,156.62) = 18.34, *p*<.001, *η* ^2^ = .24. prosocial and antisocial faces were recognized faster than neutral and new faces (*p* = .007, Figure 3b).

Recall task: As observed with the recognition task, recall of faces also varied with tags, *F*(2,118) = 10.61, *p*<.001, *η* ^2^ = .15. Note that performance at chance level here is at around 33%. Accuracy to prosocial and antisocial faces was similar, but better than neutral faces (Figure 3a). RT for recall also varied with tag, *F*(2,118) = 6.70, *p* = .002, *η* ^2^ = .10. Although accuracy in recognition was similar for antisocial and prosocial faces, antisocial faces were recalled significantly faster than prosocial and neutral faces (*p* = .02) (Figure 3b).

#### Discussion

In the recognition task, participants' recognition of prosocial and antisocial faces was similar but still better than for neutral ones. The enhanced recognition of faces of cheaters can be advantageous to societies too [44]. The social exchange computation theory [25], [26] proposes that successful and stable social exchanges depend on an evolved brain with a cognitive ability to detect cheaters. By remembering those who are selfish or unkind to others, a decision not to help or even avoid this person in the future can be made, thus stabilizing reciprocity [16], [22].

Response latencies for antisocial faces were markedly faster than for prosocial faces, a result opposite to the one observed when the encoding scenarios referred to financial transactions. The behaviors used in antisocial tags contain activities clearly classified as criminal (*e.g.*, kidnap of a young woman), which might have led to faster reaction times, although not to a higher accuracy to antisocial faces. This explanation is supported by research which found that that priming category information increased the ease of judgment-relevant information retrieval during impression formation when no prior memory of behavior was present [45]. It is also possible that the antisocial behaviors listed in the scenario were considered more negative than the prosocial behaviors were considered positive, potentially enhancing response latencies due to negativity biases in person perception [46]. The difference in response latency between the groups of faces might have occurred due to differences in the saliency of words linked to antisocial behaviors (e.g., illegal, invaded, fraud, kidnapped) in comparison to words linked to prosocial behaviors (e.g., donated, charity, helped, fostered) and neutral behaviors (e.g. hobbies, shopping, swimming, eating out). Although similar accuracy for faces in prosocial and antisocial groups point to similar saliency, more data is necessary to confirm this finding.

## Discussion

The results reported here show that recognition biases, measured as accuracy and response latency, are modulated by the social context and correspondent behavioral descriptors tagged to faces during encoding. Control experiments confirmed that recognition of faces encoded in the absence of such descriptors was similar. Experiments 1 and 2 used a financial encoding scenario and revealed an enhanced recognition of prosocial faces when the moral status of the behavioral descriptors tagged to faces was not explicit (e.g., the words “cooperators” and “cheaters” were absent and rules of social contract were not stated). The higher accuracy for faces tagged with prosocial behavior was accompanied by shorter response latencies, *i.e.*, participants recognized prosocial faces better and faster than antisocial or neutral faces. Experiment 3 showed that such bias could be eliminated, or significantly attenuated, when the moral status was made explicit by adding the words “cooperators”, “cheaters”, and “neutrals” to the descriptors and by making participants aware of the importance they assign to identifying people who display cooperative, cheating and neutral/indifferent moral behaviors via a pre-test questionnaire. Finally, experiment 4 showed that accuracy for prosocial and antisocial faces was similar but higher than for neutral faces in a social judgment encoding scenario, which contained a range of descriptors with salient moral status. Response latency to antisocial faces in such encoding scenario was *faster* than response latency to prosocial and neutral faces.

The speed and accuracy of judgments like trustworthiness can be impaired by incongruent information about traits and the behavioral events in which they occurred [45]. Experiment 4, however, offered quick access to relevant information about the moral status of faces and the context in which they appeared. The faster latencies observed for antisocial behaviors in this experiment may be explained by the finding that negative (and extreme) rather than positive (and moderate) moral behaviors are considered more diagnostic of trait behavior, whereas positive rather than negative competency-related behaviors are considered more diagnostic [47]. Participants may therefore have assigned a moral category to faces associated with antisocial-immoral rather than prosocial-moral behaviors more quickly in this experiment, making recognition of these faces faster.

As mentioned previously, it is important to remember faces of cheaters and cooperators alike to know who to approach and who to avoid in any future instance of social exchange [31], [42]. Barclay [48] suggested that biases in face recognition were modulated by the frequency of faces tagged as cheaters or cooperators in the sample; cheater recognition would be enhanced when they were the minority but it would declined when they were the majority. When the proportion of cheaters and cooperators was the same, a bias towards cooperators would emerge, in agreement with our results in experiments 1 and 2. The relative frequency of cheaters and cooperators, however, is not enough to explain the range of biases reported herein and in early studies. The financial scenario in experiments 1 and 2 was adapted from one of the scenarios used by Chiappe and colleagues [30] where the rules of the social contract and correspondent moral status were added to the descriptors tagged to faces. They reported longer response latencies and better accuracy for faces of cheaters and interpreted the results as supporting a cheater detection mechanism. The pattern of results showed a trade-off between accuracy (high) and response latency (longer). We also found longer response latencies for faces of cheaters than cooperators, but accuracy to cheaters was *lower* than for cooperators (i.e., no trade-offs). The disparity in results might be explained by the absence of statements about rules of social contract in the descriptors in this study. This idea is reinforced by the absence of memory biases in experiment 3 due to the effect of a pre-test questionnaire and words in the descriptors making explicit reference to cheating, cooperation and neutral/indifferent behaviors. This is in line with more recent studies reporting an absence of biases in face recognition [31], [42].

Variations in experimental setup might be responsible for some of the discrepancies between this and early studies where participants had to complete different tasks while memorizing the faces (e.g., attractiveness ratings), the interval for memorization was often unrestricted, the moral status of behaviors was made explicit during encoding, and accuracy was usually capped at high values (usually >90%) [27], [29], [31], [42], [49]. In our experiments a wider range of accuracies was analyzed (>60% in modified yes-no and forced-choice tasks or >33% in recall tasks) and accuracy for each participant was the average of 3–4 trials/face/descriptor, conferring robustness to the final accuracy values used in statistical analysis.

Todorov and colleagues [50] found that people were better at categorizing faces which were associated with nice behaviors, than faces associated with aggressive or disgusting behaviors, and that faces associated with positive or negative behaviors were easier to categorize than those associated neutral behaviors. Faces associated with prosocial or antisocial behaviors evoked a stronger response in particular brain regions (e.g., the anterior paracingulate cortex and areas of the superior temporal sulcus) than faces that were not associated with behaviors. Singer and colleagues [23] showed strong activation of areas primarily involved in the processing of socially relevant information when faces of cooperators were presented. The amygdala, a subcortical brain region vital for fear conditioning and consolidation of emotional memories [51], has also been linked to the assessment of face trustworthiness [24], [52], [53]. These findings support our conclusion that face processing and recognition is highly dependent on the social context and associated behavioral information encoded.

Biases in face recognition may emerge in some scenarios of social exchange if the rules of social contract and behavioral moral status are ambiguous. A transactional relationship between face recognition and social context (with its descriptors, social contract rules, social diversity, and relative frequency of cheaters and cooperators) needs to be established in order to predict recognition biases in different scenarios of social exchange. The results described here showed that the presence or absence of statements about the moral status and rules of social contract in descriptors tagged to learned faces led to diverging results. In the absence of such information, participants tended to show an enhanced recognition of cooperators. Furthermore, in diverse social scenarios, individuals in a community under the influence of social norms tend to cooperate even in the absence of mechanisms based on punishment [54]. Taken together, the biases in recognition reported here show that far from being narrowly customized to a fixed type of response, face recognition employs broad and flexible cognitive mechanisms finely tuned to minor changes in the content of social and behavioral information encoded with faces.

## References

[1] Bruce V, Young A (1986). Understanding face recognition.. British Journal of Psychology.

[2] Blais C, Jack RE, Scheepers C, Fiset D, Caldara R (2008). Culture Shapes How We Look at Faces.. PLoS ONE.

[3] Bonner L, Burton AM, Bruce V (2003). Getting to know you: how we learn new faces.. Visual Cognition.

[4] Willis J, Todorov A (2006). First impressions. Making up your mind after a 100-ms exposure to a face.. Psychological Science.

[5] Todorov A, Uleman JS (2003). The efficiency of binding spontaneous trait inferences to actors_ faces.. Journal of Experimental Social Psychology.

[6] Wixted JT (2007). Dual-process theory and signal-detection theory of recognition memory.. Psychological Review.

[7] Mandler G (1980). Recognizing: The judgment of previous occurrence.. Psychological Review.

[8] Stevens JR, Cushman FA, Hauser MD (2005). Evolving the psychological mechanisms for cooperation.. Annual Review of Ecology, Evolution, and Systematics.

[9] van Vugt M, De Cremer D (1999). Collective action in social dilemmas: The impact of group identification on the selection and cooperation with leaders.. Journal of Personality and Social Psychology.

[10] van Vugt M, Hart CM (2004). Social identity as social glue: The origins of group loyalty.. Journal of Personality and Social Psychology.

[11] Cohen TR, Wildschut T, Insko CA (2010). How communication increases interpersonal cooperation in mixed-motive situations.. Journal of Experimental Social Psychology.

[12] Gino F, Ayal S, Ariely D (2009). Contagion and differentiation in unethical behavior: The effect of one bad apple on the barrel.. Psychological Science.

[13] Kerr NL, Rumble AC, Park ES, Parks CD, Ouwerkerk JW (2009). “How many bad apples does it take to spoil the whole barrel?”: Social exclusion and tolerance for bad apples.. Journal of Experimental Social Psychology.

[14] Cosmides L, Barrett HC, Tooby J (2010). Adaptive specializations, social exchange, and the evolution of human intelligence.. PNAS.

[15] Kiyonari T, Barclay P (2008). Free-riding may be thwarted by second-order rewards rather than punishment.. Journal of Personality and Social Psychology.

[16] Trivers R (1971). The evolution of reciprocal altruism.. Quarterly Review of Biology.

[17] Zhang Y, Epley N (2009). Self-centered social exchange: Differential use of costs versus benefits in prosocial reciprocity.. Journal of Personality and Social Psychology.

[18] Flynn FJ, Reagans RE, Amanatullah ET, Ames DR (2006). Helping one's way to the top: Self-monitors achieve status by helping others and knowing who helps whom.. Journal of Personality and Social Psychology.

[19] Hardy CL, van Vugt M (2006). Nice guys finish first: The competitive altruism hypothesis.. Personality and Social Psychology Bulletin.

[20] Axelrod R, Hamilton WD (1981). The evolution of cooperation.. Science.

[21] Brown WM, Moore C (2000). Is prospective altruist detection an evolved solution to the adaptive problem of subtle cheating in cooperative ventures? Evidence from the Wason Selection Task.. Evolution of Human Behavior.

[22] Brown WM, Palameta B, Moore C (2003). Are there non-verbal cues to commitment? An exploratory study using the zero-acquaintance video presentation paradigm.. Evolutionary Psychology.

[23] Singer T, Kiebel SJ, Winston JS, Dolan RJ, Frith CD (2004). Brain responses to the acquired moral status of faces.. Neuron.

[24] Winston JS, Strange BA, O'Doherty J, Dolan RJ (2002). Automatic and intentional brain responses during evaluation of trustworthiness of faces.. Nature Neuroscience.

[25] Cosmides L (1989). The logic of social exchange: Has natural selection shaped how humans reason? Studies with Wason selection task.. Cognition.

[26] Cosmides L, Tooby J, Barkow J, Cosmides L, Tooby J (1992). Cognitive adaptions for social exchange.. The adapted mind: Evolutionary psychology and the generation of culture.

[27] Farelli D, Turnbull N (2008). The role of reasoning domain on face recognition: detecting violations of social contract and hazard management rules.. Evolutionary Psychology.

[28] Laughery KR, Alexander JF, Lane AB (1971). Recognition of human faces: Effects of target exposure time, target position, pose position and type of photograph.. Journal of Applied Psychology.

[29] Mealey L, Daood C, Krage M (1996). Enhanced memory for faces of cheaters.. Ethology and Sociobiology.

[30] Chiappe D, Brown A, Dow B, Koontz J, Rodriguez M (2004). Cheaters are looked at longer and remembered better than co-operators in social exchange.. Evolutionary Psychology.

[31] Mehl B, Buchner A (2008). No enhanced memory for cheaters.. Evolution and Human Behavior.

[32] Barclay P, Lalumiere M (2006). Do people differently remember cheaters?. Human Nature.

[33] Bayley PJ, Wixted JT, Hopkins RO, Squire LR (2008). Yes/no recognition, forced-choice recognition, and the human hippocampus.. Journal of Cognitive Neuroscience.

[34] Bastin C, Van der Linden M (2003). The contribution of recollection and familiarity to recognition memory: A study of the effects of test format and aging.. Neuropsychology.

[35] Aggleton J, Shaw C (1996). Amnesia and recognition memory: A re-analysis of psychometric data.. Neuropsychologia.

[36] Jiang F, Blanz V, O'Toole AJ (2009). Three-dimensional information in face representations revealed by identity aftereffects.. Psychological Science.

[37] Felisberti FM, Aidoo B (2009). Enhanced memory for cooperators in a face-in-the-crowd task..

[38] Brown., Moore C (2000). Is prospective altruist detection an evolved solution to the adaptive problem of subtle cheating in cooperative ventures? Evidence from the Wason Selection Task.. Evolution of Human Behavior.

[39] Price ME (2006). Judgments about cooperators and freeriders on a Shuar work team: An evolutionary psychological perspective.. Organizational Behavior and Human Decision Processes.

[40] Oda R, Hiraishi K, Matsumoto-Oda A (2006). Does an altruist-detection cognitive mechanism function independently of a cheater-detection cognitive mechanism? Studies using Wason selection tasks.. Evolution and Human Behavior.

[41] Gold JJ, Smith CN, Bayley PJ, Shrager Y, Brewer JB (2006). Item memory, source memory, and the medial temporal lobe: Concordant findings from fMRI and memory-impaired patients.. Proceedings of the National Academy of Sciences.

[42] Buchner A, Bell R, Mehl B, Musch J (2009). No enhanced recognition memory, but better source memory for faces of cheaters.. Evolution and Human Behavior.

[43] Komorita SS, Parks CD, Hulbert LG (1992). Reciprocity and the induction of cooperation in social dilemmas.. Journal of Personality and Social Psychology.

[44] Shinada M, Yamagishi T (2007). Punishing free riders: direct and indirect promotion of cooperation.. Evolution and Human Behavior.

[45] Carlston DE, Skowronski JJ (1986). Trait memory and behavior memory: The effects of alternative pathways on impression judgment response times.. Journal of Personality and Social Psychology.

[46] Reeder GD, Brewer MB (1979). A schematic model of dispositional attribution in interpersonal perception.. Psychological Review.

[47] Skowronski JJ, Carlston DE (1989). Negativity and extremity biases in impression formation: A review of explanations.. Psychological Bulletin.

[48] Barclay P (2008). Enhanced recognition of defectors depends on their rarity.. Cognition.

[49] Gigerenzer G, Hug K (1992). Domain-specific reasoning: social contracts, cheating, and perspective change.. Cognition.

[50] Todorov A, Gobbini MI, Evans KK, Haxby JV (2007). Spontaneous retrieval of affective person knowledge in face perception.. Neuropsychologia.

[51] Phelps EA, LeDoux JE (2005). Contributions of the amygdala to emotion processing: From animal models to human behavior.. Neuron.

[52] Adolphs R, Tranel D, Damasio AR (1998). The human amygdala in social judgment.. Nature.

[53] Engell AD, Haxby JV, Todorov A (2007). Implicit trustworthiness decisions: Automatic coding of face properties in the human amygdala.. Journal of Cognitive Neuroscience.

[54] Santos FC, Santos MD, Pacheco JM (2008). Social diversity promotes the emergence of cooperation in public goods games.. Nature.

